# Efficacy of an unmodified bivalent mRNA vaccine against SARS-CoV-2 variants in female small animal models

**DOI:** 10.1038/s41467-023-36110-1

**Published:** 2023-02-13

**Authors:** Björn Corleis, Donata Hoffmann, Susanne Rauch, Charlie Fricke, Nicole Roth, Janina Gergen, Kristina Kovacikova, Kore Schlottau, Nico Joel Halwe, Lorenz Ulrich, Jacob Schön, Kerstin Wernike, Marek Widera, Sandra Ciesek, Stefan O. Mueller, Thomas C. Mettenleiter, Domenico Maione, Benjamin Petsch, Martin Beer, Anca Dorhoi

**Affiliations:** 1 Institute of Immunology, Friedrich-Loeffler-Institut, Greifswald-Insel Riems, Germany; 2 Institute of Diagnostic Virology, Friedrich-Loeffler-Institut, Greifswald-Insel Riems, Germany; 3 CureVac SE, Tübingen, Germany; 4 Institute for Med. Virology, University Hospital Frankfurt, Goethe University Frankfurt, Frankfurt am Main, Germany; 5 German Center for Infection Research (DZIF), Braunschweig, Germany; 6 Branch Translational Medicine and Pharmacology, Fraunhofer Institute for Molecular Biology and Applied Ecology (IME), Frankfurt am Main, Germany; 7 Friedrich-Loeffler-Institut, Federal Research Institute for Animal Health, Greifswald-Insel Riems, Germany; 8 GSK, Siena, Italy

**Keywords:** RNA vaccines, SARS-CoV-2

## Abstract

Combining optimized spike (S) protein-encoding mRNA vaccines to target multiple SARS-CoV-2 variants could improve control of the COVID-19 pandemic. We compare monovalent and bivalent mRNA vaccines encoding B.1.351 (Beta) and/or B.1.617.2 (Delta) SARS-CoV-2 S-protein in a transgenic mouse and a Wistar rat model. The blended low-dose bivalent mRNA vaccine contains half the mRNA of each respective monovalent vaccine, but induces comparable neutralizing antibody titres, enrichment of lung-resident memory CD8^+^ T cells, antigen-specific CD4^+^ and CD8^+^ responses, and protects transgenic female mice from SARS-CoV-2 lethality. The bivalent mRNA vaccine significantly reduces viral replication in both Beta- and Delta-challenged mice. Sera from bivalent mRNA vaccine immunized female Wistar rats also contain neutralizing antibodies against the B.1.1.529 (Omicron BA.1 and BA.5) variants. These data suggest that low-dose and fit-for-purpose multivalent mRNA vaccines encoding distinct S-proteins are feasible approaches for extending the coverage of vaccines for emerging and co-circulating SARS-CoV-2 variants.

## Abstract (web summary)

Here the authors show efficacy of a low-dose, unmodified, bivalent mRNA vaccine against SARS-CoV-2 variants in two female rodent models and find that combination of mRNA encoding Beta and Delta Spike sequences induces broadly neutralizing antibodies and robust T-cell responses.

## Introduction

Effective vaccines are critical for the control of the COVID-19 pandemic, especially as nations begin to scale back non-pharmaceutical interventions such as social distancing, travel restrictions, and isolation (https://www.ecdc.europa.eu/en/covid-19/prevention-and-control/vaccines). Since the beginning of the pandemic, new SARS-CoV-2 variants, including clinically relevant variants of concern (VOCs) have appeared, each characterized by different virulence, transmissibility, and immune escape, resulting in differences in the effectiveness of public health measures, diagnostics, vaccines, or therapeutics. While B.1.1.7 (Alpha) and B.1.617.2 (Delta) spread rapidly, particularly in the immunologically naïve population, B.1.351 (Beta) and especially B.1.1.529 (Omicron) are notable for immune escape (https://www.who.int/en/activities/tracking-SARS-CoV-2-variants). Several VOCs which have mutated, such as Beta^1^ and Omicron^2^, evade humoral responses elicited by vaccines based on ancestral S-protein sequences^3^. As a result, Omicron has quickly become globally prevalent, despite high immunization rates. Unfortunately, while Omicron appears to cause less severe disease than other variants^4^, it does not induce relevant cross-protective neutralizing antibody (nAb) titres in SARS-CoV-2 naïve populations, meaning they may be less protected against future infection compared with those previously exposed to other variants or vaccinated^5^.

The evolution of further VOCs is unpredictable; however, it is likely that new escape variants will emerge. Therefore, developing effective vaccines and vaccine strategies will remain essential^6^. SARS-CoV-2 vaccine development should benefit from the knowledge acquired with licensed (https://www.ema.europa.eu/en/human-regulatory/overview/public-health-threats/coronavirus-disease-covid-19/treatments-vaccines/vaccines-covid-19/covid-19-vaccines-authorised) and exploratory vaccines, to aid optimization and to broaden protection against different VOCs. For example, prototypes of multivalent nanoparticle vaccines have been reported to induce broad reactivity against different sarbecoviruses^7^. mRNA vaccines are promising candidates for future vaccine approaches, based on their demonstrated ability to induce robust protection against SARS-CoV-2^8^. The development of low-dose multivalent human vaccine preparations against different VOCs is an innovative approach^9^.

Previously designed unmodified mRNA vaccines encoding the S-protein sequences from the ancestral strain (CV2CoV and CVnCoV) have been thoroughly assessed and compared in preclinical models. CV2CoV has now entered clinical testing (NCT05260437) and has been shown to induce high neutralizing antibodies in rats^10^ and non-human primates^11^, with titers comparable to those seen with Comirnaty, a licensed mRNA COVID-19 vaccine. Protection induced by CV2CoV was reported to be superior to CVnCoV in a non-human primate SARS-CoV-2 challenge model^11^.

In this study, we tested a bivalent, optimized, unmodified mRNA vaccine candidate in two rodent models: demonstrating both feasibility and an increase in the breadth of induced immune responses against emerging VOCs. In addition, the role of T-cell dependent immunity induced by immunization with unmodified monovalent and bivalent mRNA vaccines was further elucidated.

## Results

We designed unmodified mRNA vaccines encoding the S-protein sequences from the Beta (CV2CoV.351) and Delta (CV2CoV.617) variants, which are distant variants with non-overlapping mutations in the receptor binding domain (RBD), as well as the ancestral strain (CV2CoV)^2,12^. Each vaccine dose contained a total of 0.5 µg mRNA and was administered intramuscularly to K18-hACE2 transgenic mice; the bivalent vaccine (CV2CoV.351 and CV2CoV.617 blended together) contained 0.25 µg mRNA of each variant, i.e., half of the amount of the monovalent vaccines. This dosage was based on previous findings from comparative preclinical evaluations of CVnCoV and the current optimized candidate vaccine CV2CoV^13^, whereby 0.5 µg was the lowest tested dose to fully protect against lethality and induce abundant neutralizing antibodies (Fig. S1a) and induce detectable spike-specific interferon γ (IFNγ) producing CD8^+^ cells (Fig. S1b). Lowering the CV2CoV dose to 0.1 µg mRNA reduced levels of neutralizing antibodies and protected only 50% of challenged mice (Fig. S1a, c), which is in line with previous studies using modified mRNA for immunization^14^.

**Fig. 1:**
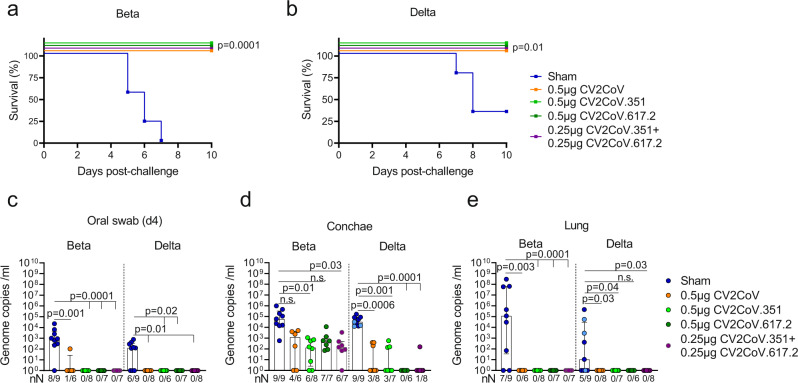
Monovalent and bivalent mRNA vaccines encoding ancestral, Beta and Delta derived S-protein sequences protect against SARS-CoV-2 variants in a transgenic mouse model. Female K18-hACE2 mice vaccinated on days 0 and 28 with a total of 0.5 µg CV2CoV (ancestral, orange), 0.5 µg CV2CoV.351 (Beta, light green), 0.5 µg CV2CoV.617.2 (Delta, dark green), CV2CoV.351 + CV2CoV.617.2 (0.25 µg of each; purple) or NaCl (sham; blue) were challenged i.n. with 10^4.4^ TCID_50_ SARS-CoV-2 variant B.1.351 (Beta) or B.1.617.2 (Delta) at day 56. Animal numbers analysed are summarized in Table S1. **a**, **b** Survival curves (Kaplan–Meier) for K18-hACE2 mice challenged with B.1.351 (Beta) (**a**) or B.1.617.2 (Delta) (**b**) with follow-up for 10 days post challenge. **c**–**e** RT-qPCR results from Day 4 oral swabs **(c)** or Day 10 conchae (**d**) and lung (**e**). Sham group samples were obtained at Day 10 (light blue) or at the humane endpoint (dark blue). Number of RT-qPCR positive and total number of animal sample are shown on the *x*-axis. Each dot represents one individual mouse. Scatter plots are labelled with median and interquartile range. *p-*values were determined by two-sided log-rank (Mantel-Cox) test **(a**, **b**) or one-way ANOVA and Dunn’s multiple comparison test (**c–e**). Differences were considered significant at *p* < 0.05 with exact *p*-values shown. Source data are provided as a Source Data file.

K18-hACE2 transgenic mice received 20 µL of a low dose^15^ monovalent CV2CoV, CV2CoV.351, CV2CoV.617 or bivalent vaccine (CV2CoV.351 and CV2CoV.617) containing a total of 0.5 µg mRNA or NaCl (sham) on Day 0 and Day 28 (Fig. S2). These transgenic mice have a human ACE2 receptor which is the major cell entry receptor for SARS-CoV-2^16,17^. Following challenge with Beta and Delta variants (10^4.4^ TCID_50_) on Day 56, all vaccinated mice were protected from SARS-CoV-2-induced lethality and virus spread, while all Beta-challenged and 67% of Delta-challenged sham-vaccinated animals succumbed to infection (Fig. 1a, b and Fig. S3a, b).

We were unable to detect SARS-CoV-2 genomic RNA (Fig. 1c–e and Fig. S3c, d) or subgenomic RNA (sgRNA) (Tables S2 and S3) in oral swabs collected on Day 4, or in lung, cerebellum and cerebrum samples taken on Day 10 in all but one of the Beta-challenged, bivalent-vaccinated animals, indicating that productive infection was prevented. The suppression of viral replication in the upper respiratory tract (URT) induced by the monovalent vaccines differed depending on the challenge virus. While the bivalent vaccine reduced viral load in the conchae, equivalent to that observed with the matched monovalent vaccine after Beta challenge (Fig. 1d), replication of the Delta variant in the conchae was abolished (no detectable sgRNA) in all vaccinated groups (Table S3). Thus, although all vaccines induced protection against SARS-CoV-2 lethality, the blended bivalent vaccine provided protection against viral replication in URT with half of the dose of each construct found in the monovalent vaccines.

**Fig. 2:**
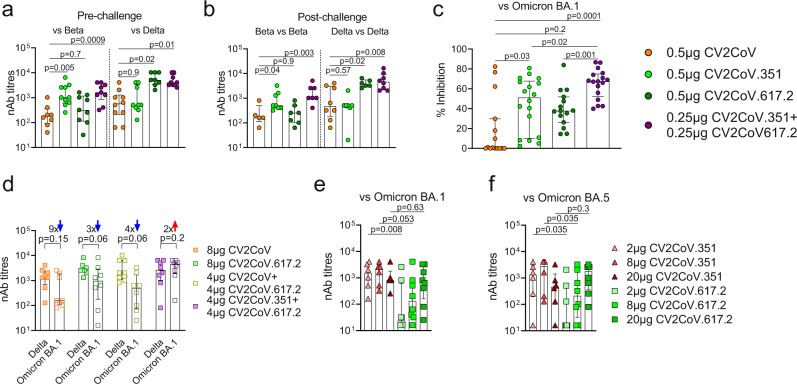
Bivalent SARS-CoV-2 mRNA vaccines induce abundant virus neutralizing titres. **a**–**c** Mice were vaccinated as described in Fig. S2, 0.5 µg CV2CoV (ancestral, orange), 0.5 µg CV2CoV.351 (Beta, light green), 0.5 µg CV2CoV.617.2 (Delta, dark green), or CV2CoV.351 + CV2CoV.617.2 (0.25 µg of each; purple). Animal numbers analysed are summarized in table S1. Neutralizing antibody (nAb) titres at Day 56 (Pre-challenge) (**a**), Day 66 (Post-challenge) (**b**), and surrogate virus-neutralization titres (sVNT) (% inhibition) at Day 56 (Pre-challenge) (**c**). Each dot represents an individual animal. Scatter plots are labelled with median and interquartile range. *p-*values were determined by one-way ANOVA and Dunn’s multiple comparison test against CV2CoV (orange) (**a**–**c**). **d**–**f** Wistar rats were vaccinated on Day 0 and 21 as described in Fig. S4, 8 µg CV2CoV (ancestral, light orange), 8 µg CV2CoV.617.2 (Delta, light green), CV2CoV + CV2CoV.617.2 (4 µg of each; light yellow) or CV2CoV.351 + CV2CoV.617.2 (4 µg of each; light purple) and nAb titres against B.1.617.2 (Delta) or B.1.1.529 (Omicron, BA. 1 and BA.5) were tested at Day 42, the numbers above the bars indicate the fold-differences (**d**). nAbs titres of each vaccination group against Delta versus Omicron BA.1; (*N* = 8 individual rats) (**d**) or nAbs titres from animals vaccinated with equal amounts of CV2CoV.351 versus CV2CoV.617.2 (2 µg CV2CoV.351 light red, 8 µg CV2CoV.351 red, 20 µg CV2CoV.351 dark red, 2 µg CV2CoV.617.2 light green, 8 µg CV2CoV.617.2 green, 20 µg CV2CoV.617.2 dark green) against Omicron BA.1 (**e**) or BA.5 (**f**) (*N* = 6 individual CV2CoV.351 vaccinated rats; *N* = 8 individual CV2CoV.617.2 vaccinated rats). Each dot represents an individual animal. Scatter plots are labelled with median and interquartile range. The *p*-values were determined by one-way ANOVA and Dunn’s multiple comparison test. Source data are provided as a Source Data file.

Antibodies are critical for defence against viruses and nAbs have been shown to correlate with protection against COVID-19^18,19^ to inform immunization schedules^20^. In our study, anti-RBD total immunoglobulin levels were high in all vaccinated mice, with no notable differences between groups (Fig. S3e, f). The bivalent mRNA vaccine induced similarly high nAb titres as the Beta and Delta monovalent vaccines with their respective homologous challenges (Fig. 2a, b), despite the bivalent vaccine containing half the mRNA amount of each monovalent vaccine (0.25 µg vs. 0.5 µg). Irrespective of the VOC used for the assessment of nAbs, the bivalent mRNA vaccine induced statistically significantly higher nAb titres compared to those elicited by CV2CoV; whereas nAb titres induced by the monovalent mRNA vaccines were only statistically significantly higher than CV2CoV when they were assessed using the VOC they were designed against (Fig. 2a, b). Compared with CV2CoV, the monovalent CV2CoV.351 and the bivalent vaccine induced significantly higher nAb titres in a surrogate VNT inhibition (sVNT) assay against Omicron BA.1 (Fig. 2c). In addition, nAbs titres were significantly higher for the bivalent vaccine compared with the monovalent vaccines (Fig. 2c).

Within additional experiments in a different animal model, serum from Wistar rats vaccinated with CV2CoV or CV2CoV.617.2 mRNA vaccines (monovalent; 8 µg), or vaccines combining CV2CoV617.2 with either CV2CoV or CV2CoV.351 (bivalent; 4 µg of each) on Day 0 and Day 21 (Fig. S4) had high levels of nAb titres against Delta (Fig. 2d). Including the Beta S-protein sequence in the bivalent vaccine appeared to improve the neutralizing capacity of sera against Omicron BA.1, compared with vaccines without Beta S-protein, although statistical significance was not achieved (Fig. 2d). To determine the relative contribution of CV2CoV.351 or CV2CoV.617.2 to the increased nAb titres and antigenic breadth of the bivalent vaccine, we compared nAbs titres against Omicron BA.1 and BA.5 with different amounts of the monovalent vaccines 3 weeks post second dose (Fig. 2e, f). We observed that 2 µg and 8 µg doses of CV2CoV.351 vaccination induced higher nAbs against Omicron BA.1 than CV2CoV.617, suggesting that the increased capacity of the bivalent vaccine to neutralize Omicron BA.1 was dominated by CV2CoV.351 (Fig. 2e). The same pattern was observed for nAb titres against Omicron BA.5 in sera from CV2CoV.351 vaccinated animals (Fig. 2f). No significant difference in nAb titres between CV2CoV.351 and CV2CoV.617.2 at 2 µg and 8 µg against Delta were observed (Fig. S5), and the increased breadth of nAbs with CV2CoV.351 only declined at lower concentrations (Fig. S6). Thus, the bivalent formulation, containing half the amount of each mRNA compared with the monovalent vaccines, resulted in broader antigen coverage. The presence of CV2CoV.351 in the bivalent formulation appears to be crucial for the observed broader nAb breadth. This strategy may be advantageous in case of the emergence of additional antigenic distant variants in the future.

**Fig. 3:**
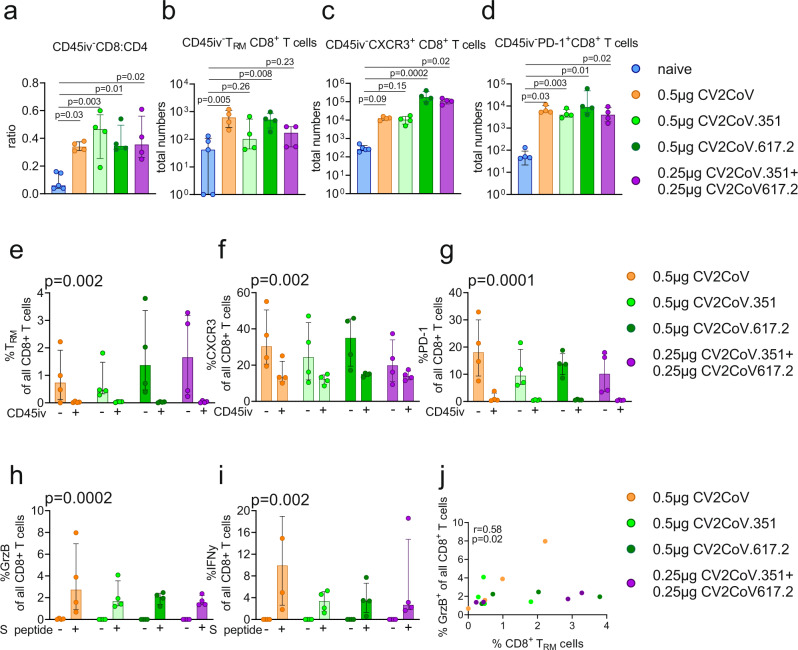
SARS-CoV-2 mRNA vaccines induce robust lung CD8^+^ T cell responses. Lung parenchyma T cells from female K18-hACE2 mice (*N* = 4 per group) at Day 56 post vaccination were investigated by in vivo injection of anti-mouse CD45 antibodies (CD45iv) prior to harvesting of lung tissue. Mice received 0.5 µg CV2CoV (ancestral, orange), 0.5 µg CV2CoV.351 (Beta, light green), 0.5 µg CV2CoV.617.2 (Delta, dark green), CV2CoV.351 + CV2CoV.617.2 (0.25 µg of each; purple) or naive (unvaccinated; blue) **a** CD8:CD4 ratio of lung CD45iv^-^ T cells. **b**–**d** Total number of CD8^+^ T_RM_ cells (CD45iv^-^CD3^+^γδTCR^-^CD8^+^CD44^high^CD62L^-^CD103^+^CD69^+^ T cells), CD45iv^-^CXCR3^+^CD8^+^ T cells and CD45iv^-^PD-1^+^CD8^+^ T cells after vaccination. **e**–**g** Frequency of CD45iv^-^ versus CD45iv^+^ CD8^+^ T_RM_ cells, CXCR3^+^CD8^+^ T cells and PD-1^+^CD8^+^ T cells after vaccination. **h**, **i** Granzyme B (GrzB^+^) production by lung CD8^+^ T cells (**h**) and IFNy production by CD8^+^ T cells (**i**) was investigated by in vitro re-stimulation of lung cells with S-peptide pools derived from ancestral SARS-CoV-2. **j** correlation of CD8^+^ T_RM_ cells and GrzB^+^ CD8^+^ T cells determined by a non-parametric spearman correlation test. **a**–**i** Scatter plots are labelled with median and interquartile range. *P-*values were determined by one-way ANOVA and Dunn’s multiple comparison test against the naïve group (**a**–**d**), by two-way ANOVA comparing CD45iv^-^ versus CD45iv^+^ (**e**–**g**), or by two-way ANOVA comparing no stimulation (−) against stimulation with S-peptide (+) (**h**, **i**). Differences were considered significant at *p* < 0.05 with exact *p*-values displayed in the figure. Source data are provided as a Source Data file.

Lung tissue resident memory (T_RM_) T cells provide an additional layer of cross-protective mucosal immune responses and therefore are desired to be induced by vaccination. We observed that both the monovalent and bivalent mRNA vaccines triggered T cell responses dominated by increased numbers and ratios of lung CD45iv^-^ CD8^+^ T cells (Fig. 3a and Fig. S8a, b). This increase was accompanied by higher numbers of T_RM_ (CD45iv^-^CD3^+^γδTCR^-^CD8^+^CD44^high^CD62L^-^CD103^+^CD69^+^ T cells) and markers associated with T_RM_ such as CXCR3 (for homing) and PD-1 (for regulation of activation of T_RM_) (Fig. 3c–d and Fig. S8b). The frequency of T_RM_^+^, CXCR3^+^ and PD-1^+^ CD8^+^ T cells was significantly upregulated in CD45iv^-^ tissue CD8^+^ T cells compared to CD45iv^+^ lung vascular CD8^+^ T cells (Fig. 3e–g). Lung CD8^+^ T cells from vaccinated animals responded to S-peptide by upregulation of IFNγ and granzyme (GrzB) (Fig. 3h, i). Our analysis did not allow detection of S-peptide specific T_RM_ cells, however, the frequency of CD45iv^-^CD8^+^ T_RM_ cells correlated with S-peptide specific GrzB^+^ lung CD8^+^ T cells (Fig. 3j). In addition, we performed a similar analysis for CD4^+^ T cells (Fig. S8). The pattern followed the observations for CD8 + T cells, however the overall increase of CD4^+^ T cells and CD4^+^ T_RM_ cells appeared lower than for CD8^+^ T cells (Fig. S8a–e). S-peptide specific IFNy^+^ CD4^+^ T cells were detectable, but did not correlate with the frequency of CD4^+^ T_RM_ cells (Fig. S8f, g). Thus, the mRNA vaccines used in this study induced lung SARS-CoV-2 specific T_RM_ T cell responses dominated by CD8^+^ cytotoxic T cells. Importantly, blending antigens in lower amounts (bivalent vaccine) maintained the quality, i.e., tissue residency and activation patterns of T cell immune responses triggered by monovalent vaccines.

We conclude that the CV2CoV.351/CV2CoV.617.2 bivalent vaccine provides similar protection against Beta and Delta as that observed with the corresponding monovalent vaccines. Furthermore, the bivalent vaccine increases the breadth of nAbs against newly emerging VOCs such as the current Omicron variants. In addition, intramuscular application of unmodified mono- and bivalent mRNA vaccines induced S-peptide specific and T_RM_ CD8^+^ T cell responses at mucosal sites.

## Discussion

Our study demonstrated that an unmodified, blended bivalent mRNA vaccine is fully protective in transgenic mice and, collectively with additional rat studies, we have elucidated the induced humoral and cellular immune responses. Multivalent influenza^21^ and cytomegalovirus^22^ mRNA vaccines have shown that integration of multiple antigens can lead to robust nAb responses in pre-clinical models which correlate with protection for multiple vaccines^18,23^. In addition, modified bivalent mRNA SARS-CoV-2 vaccines elicited significantly higher nAb responses against recent SARS-CoV-2 variants and non-inferior titres against ancestral virus compared with monovalent vaccines in humans and preclinical animal models^9,24^. In line with these findings, our study demonstrated that blending mRNA coding for the spike protein of different variants, including the Beta spike protein, elicited antibodies with substantial breadth and the potential to neutralize newly emerging variants such as BA.5. Recent studies have revealed that repeated immunization extends neutralization to non-homologous variants, possibly through affinity maturation of memory B cell populations^12,25^. Following the effective virus clearance in conchae, and the potential impact on Delta transmission, it will be important to evaluate the impact of bivalent, and higher valency, mRNA vaccines on non-neutralizing antibody functions, e.g., antibody-dependent natural killer cell activation or antibody-mediated cellular phagocytosis, which are elicited by mRNA vaccines and maintained despite reductions in nAb titers over time^26^. These non-neutralizing antibodies may facilitate virus clearance like that observed in the URT of Delta-challenged mice. The characteristic drop in vaccine effectiveness in humans with new variants was not observed in the mouse model, highlighting a potential limitation of the predictive power of the model; however, both models used in our study provide complementary insights regarding the mechanism of action of the bivalent vaccine. The rat-derived data translated very well to improved immunogenicity and protective efficacy in a non-human primate challenge model for SARS-CoV-2^10,11^ and our cross-neutralization results emphasize the broad antigen coverage induced by the bivalent vaccine. Further studies using lower doses (such as 0.25 µg) of mono- and bivalent vaccine may contribute to our understanding of optimal dose ranges and compositions of fit-for-purpose bivalent vaccines.

CV2CoV is currently being investigated in healthy volunteers in a phase 1 clinical trial. Neutralizing antibodies in the sera of the participants will be assessed since they have been identified as one of the key parameters correlating with the observed protective efficacy of the first generation SARS-CoV-2 mRNA vaccine CVnCoV^27^, and a licensed COVID-19 mRNA vaccine^28^.

Our murine model provided additional insights into specific features of T cell immunity within various anatomical sites. In addition to humoral responses, cellular immunity contributes to protection against COVID-19 and recent evidence suggests that viral-vector and mRNA vaccines elicit long-lasting S-specific or nucleoprotein-specific CD4^+^ and CD8^+^ T cell responses, with broad cross-reactivity against VOCs^29,30^. While T_RM_ cells are induced by high concentration mRNA vaccines^31^; we report that lower concentrations of unmodified mono- or bivalent mRNA vaccines induced lung parenchymal tissue resident T cells. Quantification of T_RM_ cells has yielded variable results, as reported by others, using different modified mRNA vaccine constructs in a mouse model, ranging from their detection in the lungs^14^ to absence in the same tissue^15^ despite using it at much higher doses of 2–5 µg.

The accumulation of memory cells at mucosal sites is critical for the control of viral pneumonia and their presence has been reported to be associated with less severe COVID-19 symptoms^32^. CD8^+^ T cells have been shown to contribute to virological control of SARS-CoV-2 infection when antibodies titres were sub-optimal in non-human primates^33,34^. The role of the durability and specificity of T_RM_ CD8^+^ T cells in protection from disease requires further investigation using antibody-mediated depletion. Depletion of CD8^+^ T cells following immunization with mRNA vaccine, prior to SARS-CoV-2 challenge, led to different outcomes in a transgenic mouse model, possibly due to the vaccine dose used and variability in nAb concentrations^14,35^. Interest in vaccine-elicited T cell responses has increased due to immune escape by variants, such as Omicron^29^. Preservation of the epitope repertoire may be critical for defence against current and future variants and may bring substantial benefits by contributing to protection against severe disease. This feature, along with tissue residency, makes the bivalent blended mRNA vaccine, and more generally, multivalent vaccines, highly appropriate candidates for further development.

In summary, the evolution of SARS-CoV-2 is a challenge for vaccine-based strategies aimed at disease control. Our study demonstrates that a low-dose, bivalent blended, unmodified mRNA vaccine is highly efficacious in pre-clinical mouse and rat models. Results suggest that dose-sparing, multivalent vaccines, combining mRNA encoding the S-protein from variants with unrelated lineages may induce heterologous protection and thus increase the breadth of immune responses. In particular, the presence of CV2CoV.351 has a favourable impact on the observed nAb breadth. Given their exceptional flexibility for antigen formulation, mRNA vaccine platforms offer advantages regarding adaptability to circulating and emerging variants, and opportunities for designing pan-sarbecovirus vaccines.

## Methods

### Ethics

The animal experiments were evaluated and approved by the ethics committee of the State Office of Agriculture, Food safety, and Fishery in Mecklenburg—Western Pomerania (LALLF M-V: LVL MV/TSD/7221.3-1-055/20) and the State Office for Occupational Safety, Consumer Protection and Health in Brandenburg (LAVG: 2347-5-2021). All procedures using SARS-CoV-2 were carried out in approved biosafety level 3 (BSL3) facilities. Studies were performed using female animals only, as sex depending difference were considered not relevant for these proof-of-principle experiments. Previous studies did not unveil variability between sexes^10^ and manipulation of female animals reduces the biosafety risks in BSL3 during challenge infection.

### Study design

Female (8–10 weeks old at the time of vaccination) K18-hACE2 transgenic mice (B6.Cg-Tg(K18-ACE2)2Prlmn/J Charles River, Sulzfeld Germany) were maintained at 20–22 °C and a relative humidity of 45 ± 10% on a 12 h light/dark cycle, fed with commercial rodent chow (Ssniff, Soest, Germany), and provided with tap water ad libitum. The mice were vaccinated on Day 0 (prime) and Day 28 (boost) and infected (challenge) on Day 56, as detailed in Fig. S2. The animals were infected under short-term anaesthesia by isoflurane inhalation with 25 µl of either 10^4.4^ TCID_50_ SARS-CoV-2 lineage B.1.617.2 Delta (calculated from back-titration of the original material) or 10^4.4^ TCID_50_ SARS-CoV-2 lineage B.1.351 Beta (calculated from back-titration of the original material) per animal. An oral swab sample of each animal, under short-term isoflurane inhalation anaesthesia, was taken 4 days after infection. Animals with signs of severe clinical symptoms and/or body weight loss over 20% were euthanized immediately, all remaining animals were euthanized at Day 10 post infection.

RNA from mouse nasal swabs and organ samples was extracted using the NucleoMag® VETkit (Item no. 744200.1, Macherey-Nagel, Düren, Germany) in combination with a Biosprint 96 platform (Qiagen, Hilden, Germany). Each extracted sample was eluted in 100 µl. Viral RNA genome was detected and quantified by real-time reverse transcription polymerase chain reaction (RT-qPCR) on a BioRad real-time CFX96 detection system (BioRad, Hercules, USA) and analysed using the Biorad CFX Maestro version 2 and Microsoft Office Professional Plus Version 1808. Target sequence for amplification was the viral RNA-dependent RNA polymerase^36^. Genome copies per µl RNA template were calculated based on a quantified standard RNA, where absolute quantification was done by the QX200 Droplet Digital PCR System in combination with the 1-Step RT-ddPCR Advanced Kit for Probes (item no. 1863004, 1863005, 1863009, 1864021, BioRad, Hercules, USA). The limit of detection was calculated to be 1 genome copy/µl RNA. Samples (mouse swabs/organs) that tested positive for viral genomic RNA were evaluated using an assay specifically detecting sgRNA of the ORF7a as previously described by Hoffmann et al.^13^. Briefly, using the primers sgRNA-Lead-2F (CCA GGT AAC AAA CCA ACC AAC T), sgRNA-ORF7a- 2R (ACC TCT AAC ACA CTC TTG GTA G) and the probe sgRNA-ORF7a- 2FAM (FAM-TCT TGG CAC TGA TAA CAC TCG CTA CT-BHQ1) the RT-qPCR reaction was prepared using the qScript XLT One-Step RT-qPCR ToughMix (QuantaBio, Beverly, MA, USA) in a volume of 12.5 µl including 1 µl of the primer probe mix and 2.5 µl of extracted RNA. The reaction was performed for 10 min at 50 °C for reverse transcription, 1 min at 95 °C for activation, and 42 cycles of 10 s at 95 °C for denaturation, 10 s at 60 °C for annealing and 20 sec at 68 °C for elongation. Fluorescence was measured during the annealing phase. The RT-qPCR assay was performed on the BioRad real-time CFX96 detection system.

Female (7–8 weeks old at the time of vaccination), Wistar rats, provided and handled by Preclinics (Potsdam, Germany), were kept in Macrolon Type IV cages on 12 h light/dark cycle, at 20–22 °C and a relative humidity of 45–65%, and had unlimited access to standard diet (Ssniff R/M, Soest, Germany) and water. Animals were vaccinated on Day 0 (prime) and Day 21 (booster), as detailed in Fig. S4.

All Data of this study were visualized using GraphPad Prism Version 8.4.2.

### Vaccine

The optimised non-coding regions of mRNA SARS-CoV-2 vaccine CV2CoV have previously been shown to improve homologous and heterologous neutralising antibody responses in rats and non-human primates, compared with CVnCoV and other modified mRNA vaccines^10,11^. The mRNA CV2CoV vaccine is based on the RNActive® platform (claimed and described in e.g. WO2002098443 and WO2012019780) and is comprised of a 5′ cap1 structure a 5′ untranslated region (UTR) from the human hydroxysteroid 17-beta dehydrogenase 4 gene (HSD17B4), a GC-enriched open reading frame, a 3′ UTR from the human proteasome 20S subunit beta 3 gene (PSMB3) followed by a histone stem loop and a polyA stretch and does not include chemically modified nucleosides, as previously described^10^. The mRNA was encapsulated using the lipid nanoparticle (LNP) technology of Acuitas Therapeutics (Vancouver, Canada). The LNPs used in this study are particles of ionizable amino lipid, phospholipid, cholesterol and a PEGylated lipid. The mRNA encoded protein is based on the S-protein of SARS-CoV-2 NCBI Reference Sequence NC_045512.2 https://www.ncbi.nlm.nih.gov/nuccore/NC_045512.2, GenBank accession number YP_009724390.1 https://www.ncbi.nlm.nih.gov/protein/YP_009724390.1 and encodes for full length S featuring K986P and V987P mutations.

### SARS-CoV-2 propagation and handling

SARS-CoV-2 B.1.617.2-lineage hCoV-19/Switzerland/BE-IFIK-918-4879/2021 (GISAID accession EPI_ISL_1760647, doi:10.17616/R3Q59F) “Delta” was kindly provided by Ronald Dijkman, Institute for Infectious Diseases, University of Bern, Switzerland. SARS-CoV-2 hCoV-19/Germany/NW-RKI-I-0029/2020 B.1.351-lineage (GISAID accession EPI_ISL_803957, doi:10.17616/R3Q59F) “Beta” was kindly provided by Robert-Koch-Institut, Berlin, Germany. SARS-CoV-2 B.1.1.529 sublineage BA.1 “Omicron” FFM-ZAF0396/2021; GenBank accession: OM617939.1, GISAID accession EPI_ISL_6959868^37^ doi:10.17616/R3Q59F and sublineage BA.5 hCoV-19/South Africa/CERI-KRISP-K040013/2022, GISAID accession EPI_ISL_ 12268493 were used for virus neutralization assay. Virus stocks were propagated (three passages for Delta, two passages for Beta and Omicron) on Vero E6 cells (Collection of Cell Lines in Veterinary Medicine CCLV-RIE 0929, originating from ATCC “Vero C1008”) using a mixture of equal volumes of Eagle MEM (Hanks’ balanced salts solution) and Eagle MEM (Earle’s balanced salts solution) supplemented with 2 mM L-Glutamine, nonessential amino acids adjusted to 850 mg/L, NaHCO_3_, 120 mg/L sodium pyruvate, 10% fetal bovine serum (FBS), pH 7.2. The virus was harvested after 72 h, titrated on Vero E6 cells and stored at –80 °C until further use.

### Serology/antibody ELISA

Antibodies reactive against the receptor binding domain (RBD) of the ancestral SARS-CoV-2 were measured using the established ELISA protocol with a 1:100 pre-dilution of serum samples as previously described^13,38^. Briefly, RBD coated or treated with the coating buffer only plates were blocked using 5% skim milk in phosphate‐buffered saline. Serum samples were incubated on the coated and uncoated wells for 1 h at room temperature. Using a multi‐species conjugate (SBVMILK; obtained from ID Screen® Schmallenberg virus Milk Indirect ELISA; IDvet) diluted 1/80 for 1 h at room temperature detection was performed after the addition of tetramethylbenzidine (TMB) substrate (IDEXX) at a wavelength of 450 nm. Between each step, the plates were washed three times with Tris‐buffered saline with Tween 20. Finally, the absorbance was calculated by subtracting the optical density (OD) measured on the uncoated wells from the values obtained from the protein‐coated wells for each respective sample.

Mice sera were screened at a 1:10 dilution using a competitive enzyme-linked immunosorbent assay used with the S-RBD HRP for omicron BA.1 (SARS-CoV-2 sVNT L00847-A and S-RBD HRP Z03730, GenScript, Rijswijk, The Netherlands) according to the manufacturer’s instructions. As indicated, a reduction in optical density (OD) of ≥30% compared to the mean OD of the negative control was considered positive for the presence of neutralizing antibodies.

To specifically evaluate the presence of virus-neutralizing antibodies in serum samples, VOC variant specific virus neutralization tests were performed. Serum samples were heat-inactivated at 56 °C for 30 min. Following this, samples were serially diluted 2-fold (in triplicates) from 1:16 to 1:4096 and incubated at 37 °C and 5% CO_2_ with 100 TCID_50_ of virus in 96-well plates for 1 h. The wells were then overlaid with 100 μL of Vero E6 cells. The plates were incubated for three days and observed for CPE, after which sample neutralization endpoint titres were determined. The highest serum dilution protecting 100% of cells from CPE was taken as the neutralization titre as described^13^.

### Flow cytometry

Vaccine-induced T cells were characterized by flow cytometry. To discriminate between parenchymal and vascular T cells, vaccinated mice received 3 µg (in PBS) of anti-mouse CD45 antibody (retro-orbital administration) for 3 min during lethal anesthesia. Spleen and lung tissue from vaccinated mice or naïve controls were harvested and kept at 4 °C before either mechanical disruption (spleen) or mechanical disruption followed by enzymatic digestion (lung: 175 µg/ml LiberaseTM [Roche] and 0.1 mg/ml DNase I in serum-free medium) to generate single cell suspensions. Erythrocyte lysis in both suspensions was performed using 1 x red blood cell (RBC) lysis buffer (BioLegend) before determining cell counts (Biorad TC20 automated cell counter). After an additional washing step, cell surface receptor staining began with Zombie UV™ Fixable dye (1:100; BioLegend) for 20 min in the dark at 4 °C followed by two washing steps. Unspecific antibody binding was blocked with TruStain FcX (anti-mouse CD16/32) solution (BioLegend) for 5 minutes at 4 °C before adding freshly prepared antibody cocktails. Cells were incubated with surface antibodies for 20 min at 4 °C in the dark, followed by two washing steps before fixation for 30 min at room temperature with 4% paraformaldehyde (PFA). SARS-CoV-2 S-peptide specific responses were investigated by culturing single cell suspensions in the presence of 0.5 µg/ml PepMix™ SARS-CoV-2 (JPT) for 15 h and brefeldin A (BioLegend) for an additional 4 h. For intracellular antibody staining cells were washed twice before fixated with intracellular fixation buffer (eBioscience, Foxp3/Transcription Factor Staining Buffer Set) and washed with 1× permeabilization buffer followed by incubation of antibody cocktails diluted in 1× permeabilization buffer. Details of the antibody clones, fluorochromes, dilutions, and fluorescent minus one (FMO) controls are provided in Table S4. Cells were stored in PBS at 4 °C in the dark for no longer than 24 h before acquisition on a BD Fortessa™ instrument using DIVA Version 9.0.1 software, and analysed using FlowJo Version 10.5.3. Details of the T cell gating strategy are presented in Fig. S7.

### Reporting summary

Further information on research design is available in the Nature Portfolio Reporting Summary linked to this article.

## Supplementary information

Supplementary Information

Reporting Summary

## Data Availability

The authors declare that the data supporting the findings of this study are available within the paper and its supplementary information files and are available from the corresponding authors upon reasonable request. All non-commercial materials generated during the current study are available from the corresponding authors under an MTA with Friedrich-Loeffler-Institute. Source data are provided with this paper.

## Source data

Source Data
