# Supplementary material for: Efficacy of an unmodified bivalent mRNA vaccine against SARS-CoV-2 variants in female small animal models
Source: Nat Commun. 2023 Feb 13;14:816. doi: 10.1038/s41467-023-36110-1 (PMC9924835; doi:10.1038/s41467-023-36110-1)
Supplement: Supplementary file 1 — Supplementary Information [file 41467_2023_36110_MOESM1_ESM.pdf]

Supplemental Tables and Figures

Fig. S1. Preliminary studies to determine optimal vaccine amounts in K18-hACE2 mice

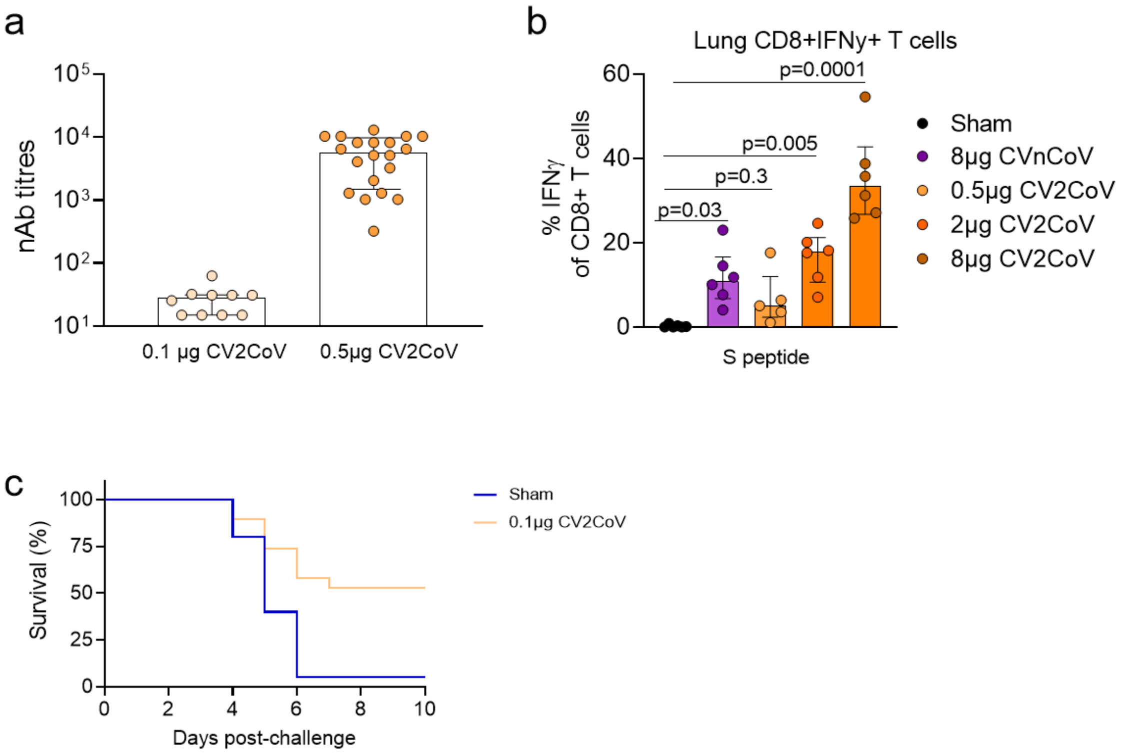

K18-hACE2 mice vaccinated on Days 0 and 28 with a total of 0.1 µg, 0.5 µg, 2 µg or 8 µg CV2CoV (ancestral, light through to dark orange), 8 µg CVnCoV (ancestral, purple) or NaCl (sham; blue). **(a)** Neutralizing antibody (nAb) titres against ancestral SARS-CoV-2 B-lineage strain BavPat1 at Day 56 (pre-challenge) as individual spots in addition to median values with interquartile range for 0.1 µg CV2CoV (ancestral, light orange, n=10) and 0.5 µg CV2CoV (ancestral, orange, n=20); results are from two independent experiments. **(b)** T cells at Day 3 post-challenge and Day 59 post-vaccination were investigated in lungs (n=5 per group; median values with interquartile range). IFNγ production by $CD8^+$ T cells was investigated by *in vitro* re-stimulation of lung cells with S-peptide pools derived from ancestral SARS-CoV-2. **(c)** Survival curves (Kaplan-Meier) for K18-hACE2 mice challenged with between 10^5 - 10^6 $TCID_{50}$ SARS-CoV-2 ancestral (BavPat1) with follow-up for 10 days post challenge (sham n=20 and 0.1µg CV2CoV n=19). P-values were determined by one-way ANOVA and Dunn's multiple comparison test against the naïve group. Differences were considered significant at $p < 0.05$ with exact p values displayed in the figure. Source data are provided as a Source Data file.

Fig. S2. Experimental design (mouse challenge experiments)

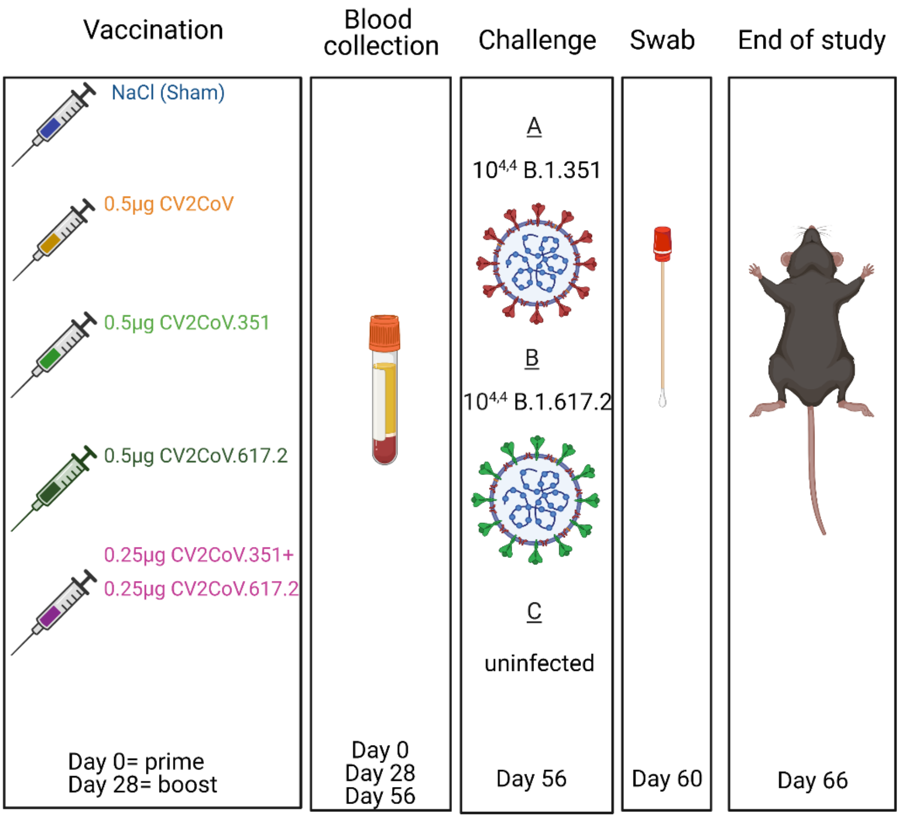

K18-hACE2 mice were vaccinated on Day 0 (prime) and Day 28 (boost) intramuscularly with 20 µl of either 0.5 µg CV2CoV (ancestral), 0.5 µg CV2CoV.351 (Beta), 0.5 µg CV2CoV.617.2 (Delta), 0.25 µg CV2CoV.351 + 0.25µg CV2CoV.617.2 (Beta and Delta) or received 20 µl NaCl (sham) as a control. Blood samples for analysis of humoral responses were collected at Day 0, 28 and 56. For the mRNA vaccinated groups, on Day 56, 4 mice per group were euthanized before viral challenge to analyse the vaccine induced cellular immune response in lung and spleen. The remaining mice were challenged with either $10^{4.4}$ B.1.351 or $10^{4.4}$ SARS-CoV-2 B.1.617.2. At 4 days post-challenge an oral swab was taken from all mice. At 10 days post-challenge, or when animals became too sick (humane endpoint), the animals were euthanized, and organs (lung, spleen, conchae, cerebrum, and cerebellum) were taken for analysis of viral load and cellular immune responses. The numbers of mice per assay are summarized in Table S1. Image was generated using the illustration software Biorender (Biorender.com).

Fig. S3. Protection against challenge with SARS-CoV-2 B.1.351 (Beta) or B.1.617.2(Delta) with monovalent and bivalent mRNA vaccines.

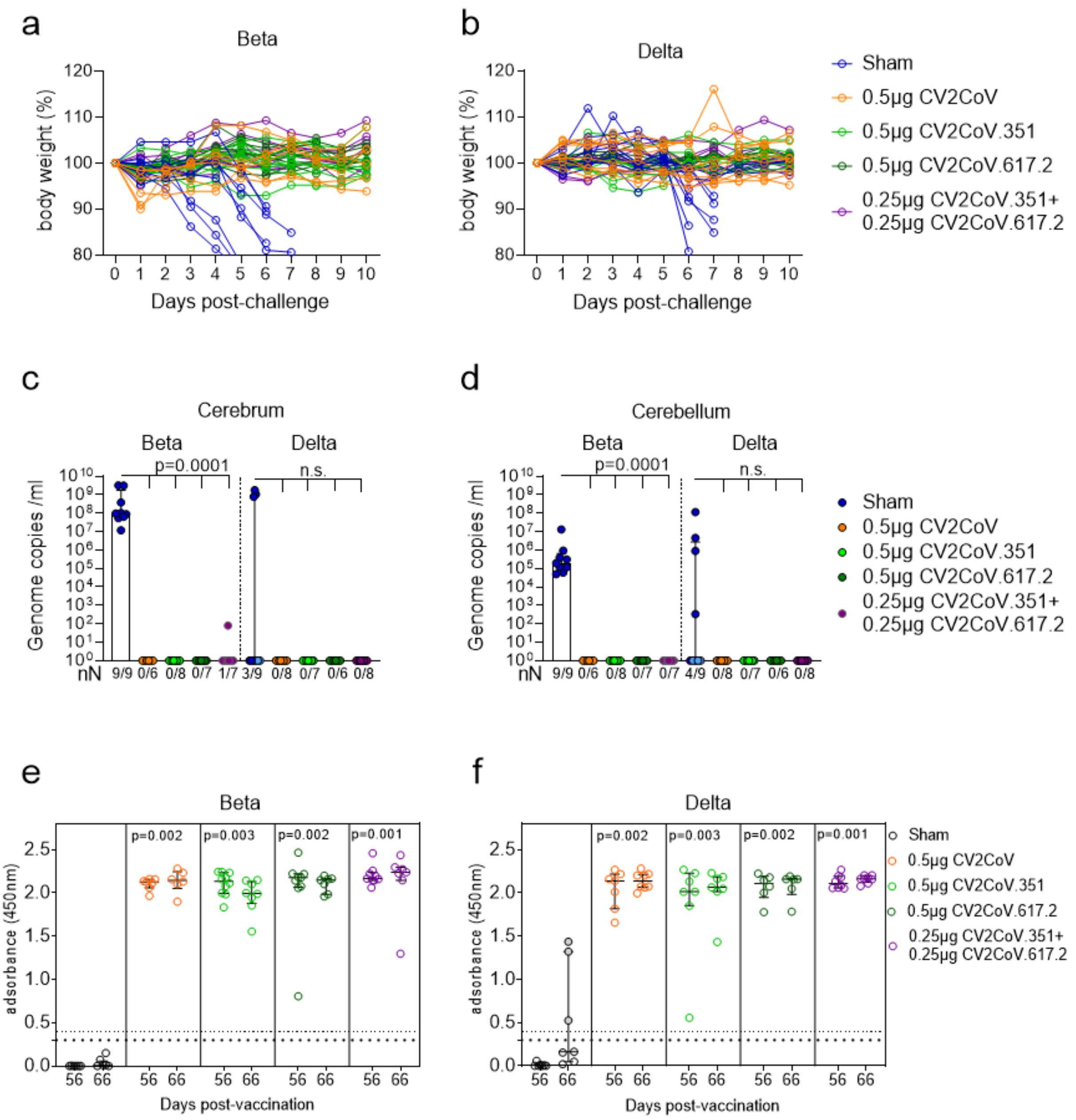

Vaccinated K18-hACE2 mice were challenged with SARS-CoV-2 variants as described in Fig. S2. The percentage change in body weight of mice infected with SARS-CoV-2 B.1.351 **(a)** or B.1.617.2 **(b)** was monitored daily. Lines represent individual animals over the course of the experiment. RT-qPCR results from Day 10 cerebrum **(c)** and cerebellum **(d)**. Sera from all vaccinated and sham animals either before challenge (Day 56) or after challenge with B.1.351 **(e)** or B.1.617.2 **(f)** (Day 66) were analysed in an RBD ELISA (ancestral RBD) for total Ig anti-RBD antibodies. Sham group samples were obtained at Day 56 or at the humane endpoint. Animal numbers analysed are summarized in table S1. Median values with interquartile range are presented. *p* Values were determined by one-way ANOVA and Dunn's multiple comparison test against the sham group (**c-f**). Source data are provided as a Source Data file.

Fig. S4. **Experimental Design (Wistar rat experiments)**

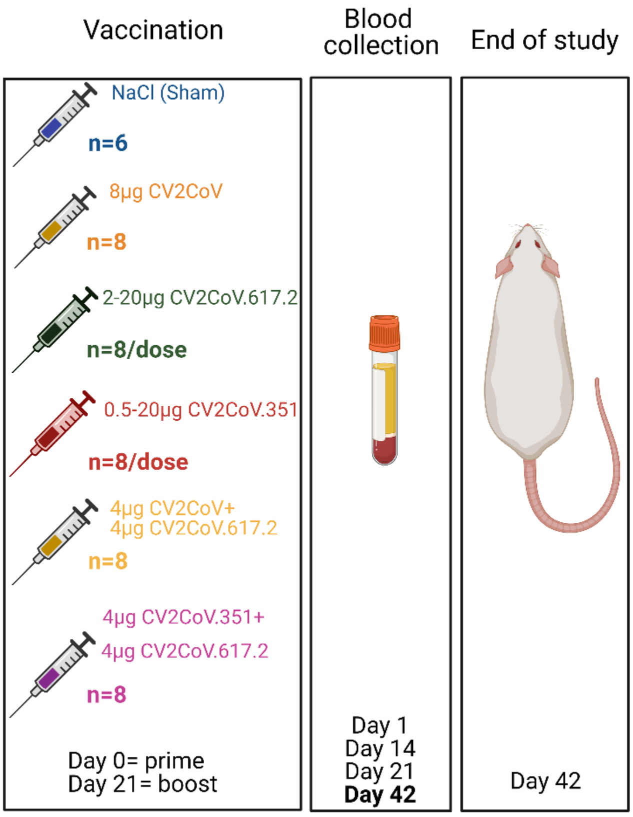

Wistar rats were vaccinated on Day 0 (prime) and Day 21 (boost) intramuscularly with either 100 µl of 8 µg CV2CoV (ancestral), 2-20 µg CV2CoV.617 (Delta), 0.5-20 µg CV2CoV.351, 4 µg CV2CoV + 4 µg CV2CoV.617 (ancestral and Delta), 4 µg CV2CoV.351 + 4 µg CV2CoV.617.2 (Beta and Delta) mRNA vaccine or received 100 µl NaCl (sham) as a control group. Blood samples were collected at Day 1, 14, 21 and Day 42. Cross-reactive responses to Omicron and Delta were only tested using Day 42 samples. The image was generated using the illustration software Biorender (Biorender.com).

Fig. S5. Potency of CV2CoV.351 compared with CV2CoV.617.2 for inducing neutralizing antibody responses against Delta in rats

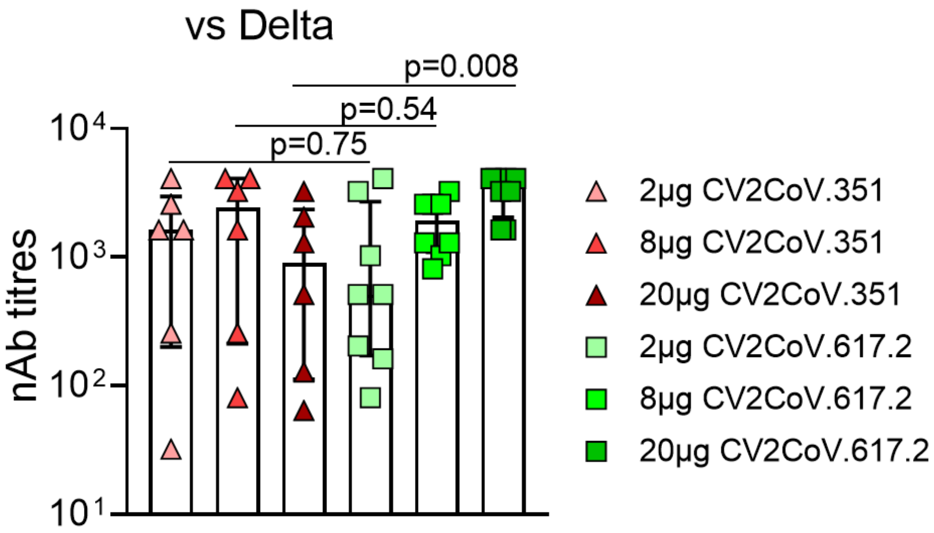

nAb titres at Day 42 (Wistar rats). Rats were vaccinated with CV2CoV.351 or CV2COV.617.2 with different concentrations as indicated, on Days 0 and 21 as described in Fig. S4 and nAb titres against Delta variant were determined. Each symbol represents an individual animal. Scatter plots are labelled with median and interquartile range. P-values were determined by one-way ANOVA and Dunn's multiple comparison test with nAbs titres from animals vaccinated with CV2CoV.351 or CV2CoV.617.2 containing the same amounts of mRNA. Source data are provided as a Source Data file.

Fig. S6. Potency of CV2CoV.351 to induce cross-neutralizing antibody responses in rats

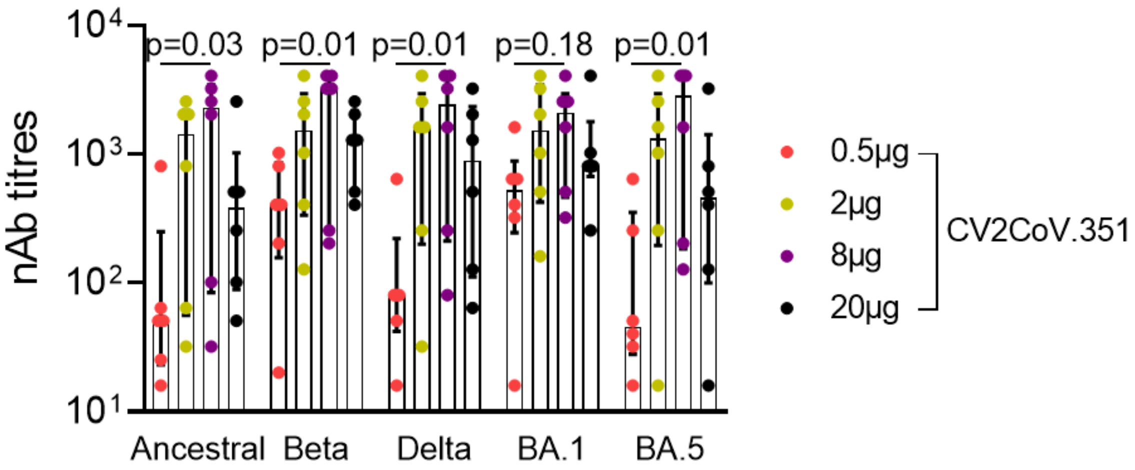

nAb titres at Day 42 (Wistar rats). Rats were vaccinated with different concentrations of CV2CoV.351, as indicated on the x-axis at Days 0 and 21 (as described in Fig. S4) and nAb titres against Ancestral, Beta, Delta, BA.1 and BA.5 variants were evaluated. Each dot represents an individual animal. The bars represent the median and vertical lines represent the interquartile range. P-values were determined by two-way ANOVA and Dunn's multiple comparison test with nAbs titres from animals vaccinated with 0.5µg CV2CoV.351 versus 2, 8 and 20µg 0.5µg CV2CoV.351. Source data are provided as a Source Data file.

Fig. S7. T-cell gating strategy

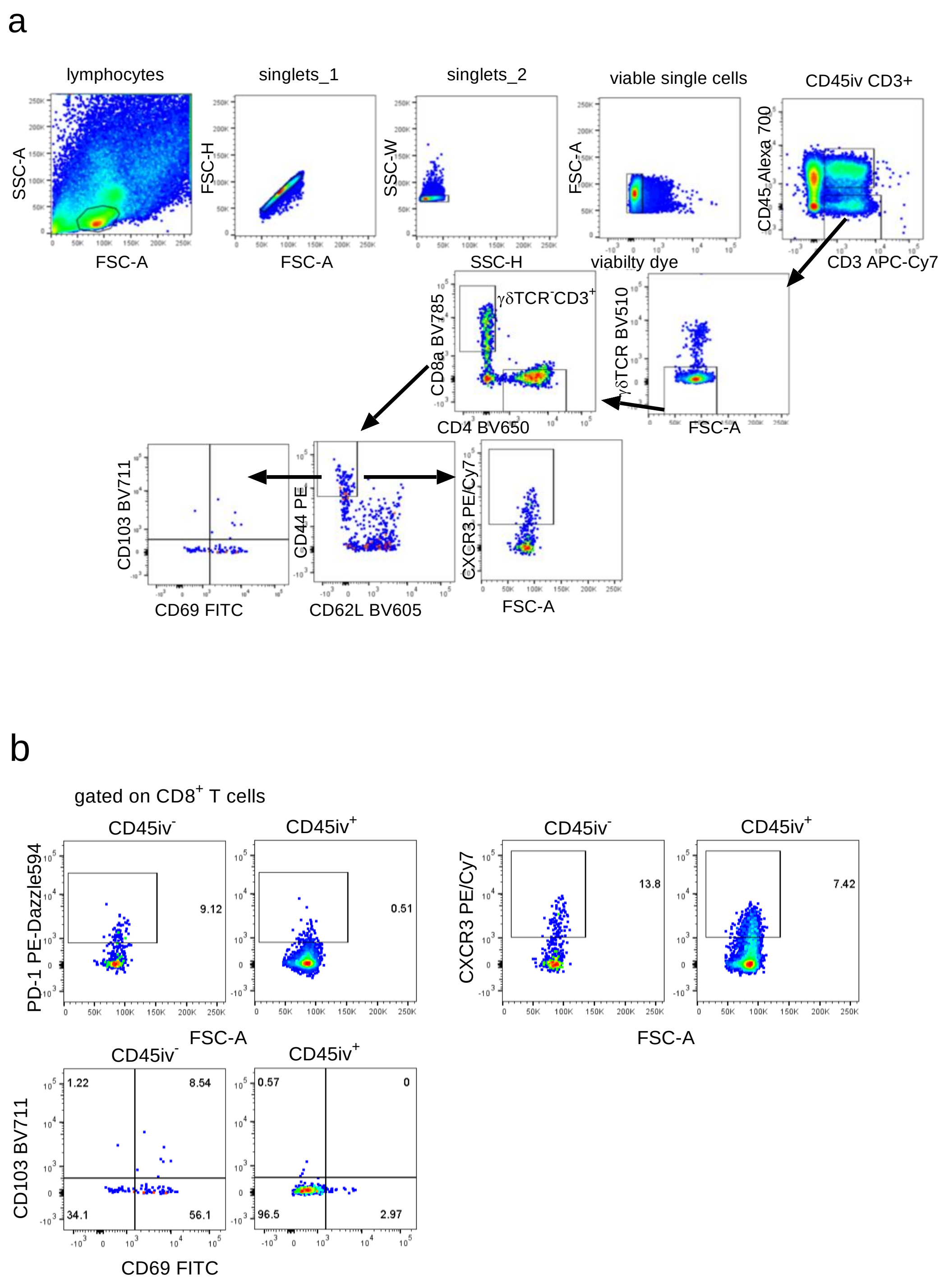

Gating strategy for T cell analysis **(a)**. Lymphocytes were identified by SSC-A vs FSC-A and lymphocyte doublets were excluded by FSC-H/FSC-A and SSC-W/SSC-H. Dead cells were eliminated using a fixable live/dead viability dye (UV zombie). To distinguish lung parenchymal (CD45iv⁻) from vascular (CD45iv⁺) CD3⁺ T cells, 3µg anti-mouse CD45 antibody was injected (i.v.) for 3 minutes during lethal anesthesia. CD3⁺ T cells were further analysed by exclusion of γδTCR⁺ cells before gating on CD8⁺ cytotoxic T cells and CD4⁺ helper T cells. CD4⁺ and CD8⁺ T cell subsets were further analysed for the

frequency of $CXCR3^+$ T cells or T_{RM} cells defined as $CD45iv^-CD3^+\gamma\delta TCR^-CD8^+CD44highCD62L^-CD103^+CD69^+$. Flow plots are generated from one representative mouse sample. Gating strategy $CD45iv^-$ versus $CD45iv^+$ T cell analysis **(b)**. T cells were analysed as described above **(a)**. $CD45iv^-$ and $CD45iv^+$ T cells were compared for their expression of markers associated with tissue residency and lung migration such as PD-1, CXCR3, CD103 and CD69. Flow plots are generated from one representative mouse sample.

Fig. S8. The induction of tissue resident T cells and S-peptide specific responses by monovalent and bivalent mRNA vaccines.

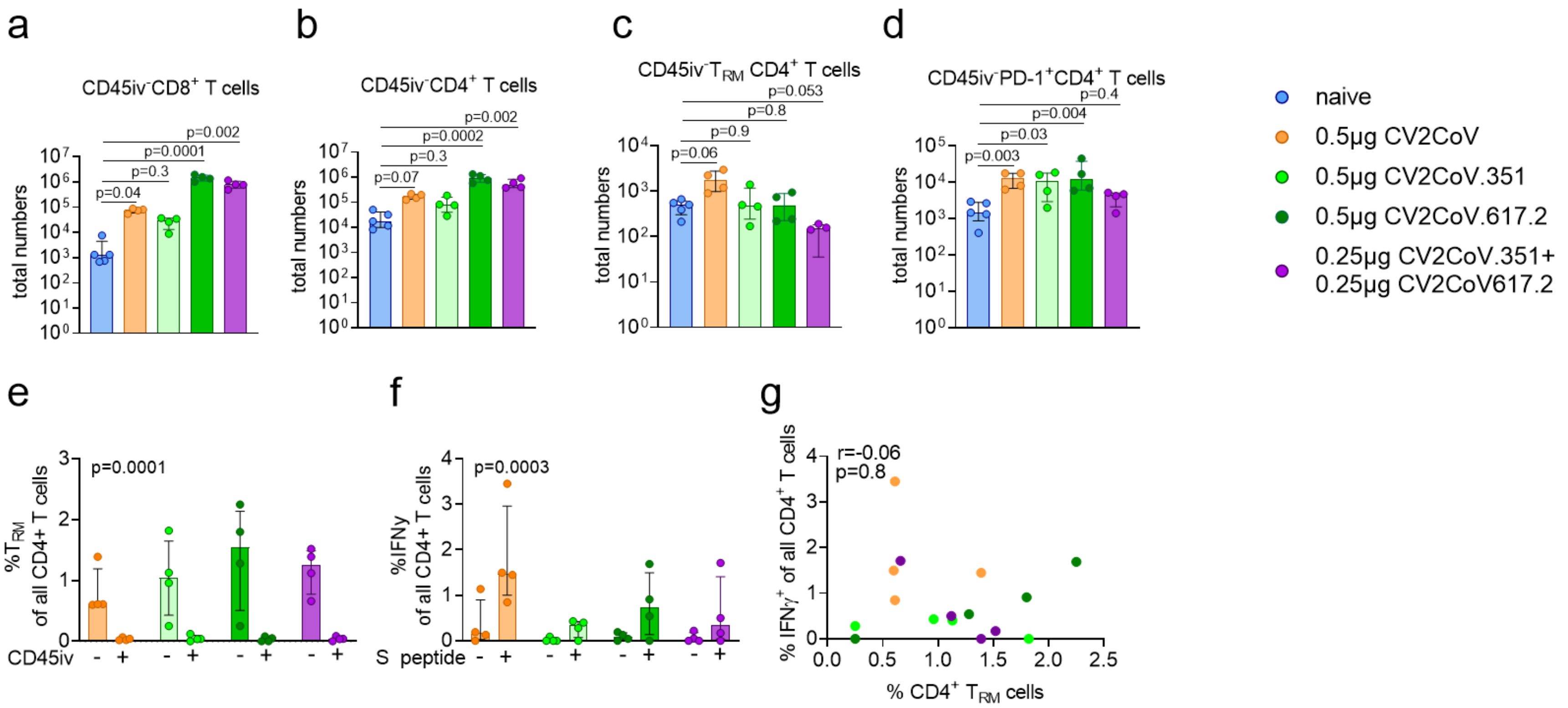

Naïve mice served as controls for analysis of T cell responses induced by monovalent and bivalent mRNA vaccines. At Day 56 post-first-dose, lung parenchyma T cells were analysed by *in vivo* injection of 3 µg anti-mouse CD45 antibodies (CD45iv) for 3 minutes before harvesting of lung tissue. Mice received 0.5 µg CV2CoV (ancestral, orange), 0.5 µg CV2CoV.351 (Beta, light green), 0.5 µg CV2CoV.617.2 (Delta, dark green), CV2CoV.351 + CV2CoV.617.2 (0.25 µg of each; purple) or NaCl (sham; blue). N=5 individual naïve mice and N=4 individual mice per vaccination regime were analysed. **(a-d)** Total number of lung $CD45iv^-$ T cells which were $CD8^+$ **(a)**, $CD4^+$ **(b)**, $CD4^+T_{RM}$ cells ($CD3^+\gamma\delta TCR^-CD4^+CD44^{high}CD62L^-CD103^+CD69^+$ T cells, **(c)**, $CD4^+PD\text{-}1^+$ T cells **(d)**. Frequency of markers associated with tissue resident (T_{RM}) cells on $CD45iv^-$ versus $CD45iv^+CD4^+$ T cells **(e)**. IFNy production by $CD4^+$ T cells was investigated by *in vitro* re-

stimulation of lung cells with S-peptide pools derived from ancestral SARS-CoV-2 **(f)**. Correlation of $CD4^+$ T_{RM} cells and $IFNy^+$ $CD4^+$ T cells determined by a non-parametric spearman correlation test (N=4 values per vaccination group) **(g)**. Each dot represents one individual mouse. Scatter plots are labelled with median and interquartile range **(a-f)**. *P v*alues were determined by one-way ANOVA and Dunn's multiple comparison test against the naïve group **(a-d)** or two-way ANOVA and Dunn's multiple comparison test comparing $CD45iv^-$ versus $CD45iv^+$ **(e)** or unstimulated (-) versus S-peptide stimulated (+) conditions **(f)**. Differences were considered significant at $p < 0.05$ with exact p values displayed in the figure. Source data are provided as a Source Data file.

Table S1. Numbers of mice per assay

Group	Total (n)	Survival data (n)	Viral loads (n)	nAb titres pre-challenge (n)	nAb titres post-challenge (n)	T cells pre-challenge (n)	RBD Elisa pre-challenge (n)	RBD Elisa post-challenge (n)
**Experiment 1 (Challenge virus: SARS-CoV-2 Beta)**								
Sham	9	9	9	N/A	N/A	N/A	9	8
CV2CoV	8	6	6	8	5	2	7	6
CV2CoV.351 (Beta)	10	8	8	10	8	2	10	8
CV2CoV.617.2 (Delta)	9	7	7	9	7	2	9	7
CV2CoV.351+CV2CoV.617.2	9	7	7	9	7	2	9	7
**Experiment 2 (Challenge virus: SARS-CoV-2 Delta)**								
Sham	9	9	9	N/A	N/A	N/A	9	7
CV2CoV	10	8	8	10	8	2	8	8
CV2CoV.351 (Beta)	9	7	7	9	7	2	7	7
CV2CoV.617.2 (Delta)	8	6	6	8	6	2	6	6
CV2CoV.351+CV2CoV.617.2	10	8	8	10	8	2	8	8
**Naive animals**								
Non-vaccinated or challenged	N/A	N/A	N/A	N/A	N/A	5	N/A	N/A

Abbreviations: N/A, not applicable; RBD, receptor-binding domain; nAB, neutralizing antibody.

Table S2. **Viral RNA genome load detected in oral swab samples Day 4 post-challenge) expressed as Cq value for total RNA and subgenomic RNA (ORF7a)**

Vaccine	Challenge	Mouse ID	Genomic RNA	Subgenomic RNA
CV2CoV	Delta B.1.617.2	25.2.3	37.6	nd
none	Beta B.1.351	24.3.3	35.0	nd
		24.3.4	29.8	36.1
		24.3.5	33.0	nd
		24.4.1	36.0	nd
		24.4.2	34.3	nd
		24.4.3	34.5	nd
		24.4.4	31.7	nd
		24.4.5	36.5	nd
	Delta B.1.617.2	25.3.1	38.0	nd
		25.3.2	38.1	nd
		25.3.3	35.4	nd
		25.4.2	36.3	nd
		25.4.4	36.8	nd
		25.4.5	37.8	nd

Abbreviation: Cq, RT-PCR cycle number at which reaction curves intersected the threshold line (also referred to as the crossing point); CV2CoV, ancestral mRNA vaccine; nd, not detected.

This table includes data for Day 4 swab samples that were positive for SARS-CoV-2 genomic RNA (Cq <45); all other Day 4 swab samples were negative (Cq ≥45). Subgenomic RNA analysis was only conducted for Day 4 swab samples that tested positive for genomic RNA.

Table S3 Viral RNA genome load detected in organ samples (10 days post challenge infection or indicated), expressed as Cq value for total RNA and subgenomic RNA (ORF7a)

Vaccine	Challenge	Mouse ID (days post-challenge)	Organ	Genomic RNA	Subgenomic RNA
none	Beta B.1.351	24.3.2 (7)	Cerebellum	21.6	23.1
			Cerebrum	14.1	15.4
			Conchae	35.5	nd
			Lung	30.0	33.2
		24.3.3 (6)	Cerebellum	26.5	28.6
			Cerebrum	19.0	20.6
			Conchae	30.4	35.4
		24.3.4 (5)	Cerebellum	25.3	25.7
			Cerebrum	17.0	18.2
			Conchae	26.3	29.7
			Lung	19.4	22.2
		24.3.5 (5)	Cerebellum	29.1	30.7
			Cerebrum	19.7	21.4
			Conchae	28.2	33.6
			Lung	17.4	20.4
		24.4.1 (7)	Cerebellum	28.2	31.5
			Cerebrum	14.1	17.1
			Conchae	30.9	35.9
			Lung	38.4	28.4
		24.4.2 (6)	Cerebellum	28.1	30.0
			Cerebrum	18.8	20.3
			Conchae	27.5	31.8
			Lung	28.2	31.2
		24.4.3 (5)	Cerebellum	26.8	28.4
			Cerebrum	19.5	21.1
			Conchae	25.2	29.0
			Lung	19.6	21.8
		24.4.4 (5)	Cerebellum	29.3	30.9
			Cerebrum	21.8	22.5
			Conchae	29.1	34.8
			Lung	26.3	29.2
		24.4.5 (6)	Cerebellum	27.5	28.6
			Cerebrum	18.9	20.5
			Conchae	31.1	35.5
CV2CoV	Beta B.1.351	24.1.3 (10)	Conchae	36.5	nd
		24.1.4 (10)	Conchae	33.0	nd
		24.1.5 (10)	Conchae	32.0	nd
		24.2.3 (10)	Conchae	32.3	nd
CV2CoV.351	Beta B.1.351	24.5.1 (10)	Conchae	34.1	nd
		24.5.2 (10)	Conchae	41.0	nd
		24.5.4 (10)	Conchae	35.0	nd
		24.6.1 (10)	Conchae	37.3	nd
		24.6.3 (10)	Conchae	34.7	nd
		24.6.5 (10)	Conchae	36.8	nd
CV2CoV.617.2	Beta B.1.351	24.7.2 (10)	Conchae	30.8	40.9
		24.7.3 (10)	Conchae	36.7	nd
		24.7.4 (10)	Conchae	34.3	nd
		24.7.5 (10)	Conchae	38.4	nd
		24.8.1 (10)	Conchae	35.6	nd
		24.8.3 (10)	Conchae	35.5	40.8
		24.8.5 (10)	Conchae	38.1	nd
		24.9.4 (10)	Conchae	38.1	nd

Vaccine	Challenge	Mouse ID (days post-challenge)	Organ	Genomic RNA	Subgenomic RNA
CV2CoV.351/ CV2CoV.617.2	Beta B.1.351	24.9.5 (10)	Conchae	38.4	nd
		24.10.1 (10)	Conchae	33.0	nd
		24.10.2 (10)	Cerebrum	37.7	nd
			Conchae	36.4	nd
		24.10.3 (10)	Conchae	36.9	nd
		24.10.5 (10)	Conchae	35.2	nd
none	Delta B.1.617.2	25.3.1 (7)	Cerebellum	22.8	25.2
			Cerebrum	14.9	18.9
			Conchae	30.4	34.5
			Lung	36.3	nd
		25.3.2 (7)	Cerebellum	25.2	28.0
			Cerebrum	14.2	16.8
			Conchae	27.6	32.1
		25.3.3 (7)	Conchae	29.2	37.2
			Lung	41.6	nd
		25.3.4 (7)	Conchae	31.3	nd
		25.4.1 (7)	Cerebellum	18.1	21.0
			Cerebrum	15.4	18.3
			Conchae	28.6	33.2
		25.4.2 (10)	Conchae	31.6	41.2
			Lung	33.7	nd
		25.4.3 (10)	Conchae	30.2	35.6
		25.4.4 (10)	Conchae	31.3	37.4
			Lung	29.4	31.9
		25.4.5 (7)	Cerebellum	36.6	nd
			Conchae	28.5	32.4
			Lung	26.2	28.9
CV2CoV	Delta B.1.617.2	25.1.4 (10)	Conchae	36.6	nd
		25.2.3 (10)	Conchae	37.5	nd
		25.2.5 (10)	Conchae	36.6	nd
CV2CoV.351	Delta B.1.617.2	25.5.1 (10)	Conchae	36.0	nd
		25.5.4 (10)	Conchae	38.1	nd
		25.6.4 (10)	Conchae	37.9	nd
CV2CoV.351/ CV2CoV.617.2	Delta B.1.617.2	25.10.3 (10)	Conchae	38.9	nd

Abbreviations: Cq, RT-PCR cycle number at which reaction curves intersected the threshold line (also referred to as the crossing point); nd, not detected.

This table includes data for organ samples that were positive for SARS-CoV-2 genomic RNA (Cq <45); all other organ samples were negative (Cq ≥45). Subgenomic RNA analysis was only conducted for organ samples that tested positive for genomic RNA.

Table S4 Antibody panels for T cell surface receptor and intracellular staining analyses in Fig. 3 and Fig. S1,8

Molecule	Fluorochrome	Isotype	Clone	Company	Cat #	Dilution factor
**Panel for T cell surface receptor analysis**						
CD45(iv)	Alexa Fluor® 700	Rat IgG2b, κ	30-F11	BioLegend GmbH	103127	3 µg in 100µl per mouse (i.v. application):
CD3	APC/Cyanine7	Rat IgG2b, κ	17A2	BioLegend GmbH	100221	100
CD4	Brilliant Violet 650™	Rat IgG2a, κ	RM4-5	BioLegend GmbH	100545	150
CD8a	Brilliant Violet 785™	Rat IgG2a, κ	53-6.7	BioLegend GmbH	100749	100
TCR γ/δ	Brilliant Violet 510™	Armenian Hamster IgG	GL3	BioLegend GmbH	118131	50
CD62L	Brilliant Violet 605™	Rat IgG2a, κ	MEL-14	BioLegend GmbH	104437	100
CD103	Brilliant Violet 711™	Armenian Hamster IgG	2E7	BioLegend GmbH	121435	100
CD95	APC	Rat IgG1, κ	SA367H8	BioLegend GmbH	152603	150
CD44	PE	Rat IgG2b, κ	IM7	BioLegend GmbH	103023	150
KLRG1	Brilliant Violet 421™	Syrian Hamster IgG	2F1/KLRG1	BioLegend GmbH	138413	100
CD183/ CXCR3	PE/ Cy7	Armenian Hamster IgG	CXCR3-173	BioLegend GmbH	126515	100
CD69	FITC	Armenian Hamster IgG	H1.2F3	BioLegend GmbH	104505	100
PD-1	PE-Dazzle 594	Rat IgG2b, κ	RMP1-30	BioLegend GmbH	109115	100
**Panel for intracellular staining of T cells after stimulation**						
CD3	APC/Cyanine7	Rat IgG2b, κ	17A2	BioLegend GmbH	100221	100
CD4	FITC	Rat IgG2a, κ	RM4-5	BioLegend GmbH	100509	100
CD8a	Brilliant Violet 785™	Rat IgG2a, κ	53-6.7	BioLegend GmbH	100749	100
TCR γ/δ	Brilliant Violet 510™	Armenian Hamster IgG	GL3	BioLegend GmbH	118131	50
T-bet	Brilliant Violet 711™	Mouse IgG1, κ	4B10	BioLegend GmbH	644819	100
RORγT	PE-CF594	Mouse IgG2a	Q31-378	BD	562684	100
FoxP3	PE-Cy5.5	Rat / IgG2a, κ	FJK-16s	ThermoFisher Scientific	35-5773-80	100
IFN-γ	Brilliant Violet 605™	Rat IgG1, κ	XMG1.2	BioLegend GmbH	505839	150
IL-17A	Brilliant Violet 421™	Rat IgG1, κ	TC11-18H10.1	BioLegend GmbH	506925	100
IL-10	PE-Cy7	Rat IgG2b, κ	JES516E3	BioLegend GmbH	505025	50

Granzyme B	Alexa Fluor® 647	Mouse IgG1, κ	GB11	BioLegend GmbH	515405	100
