# Supplementary material for: Efficacy of an unmodified bivalent mRNA vaccine against SARS-CoV-2 variants in female small animal models
Source: Nat Commun. 2023 Feb 13;14:816. doi: 10.1038/s41467-023-36110-1 (PMC9924835; doi:10.1038/s41467-023-36110-1)
Supplement: Supplementary file 2 — Reporting Summary [file 41467_2023_36110_MOESM2_ESM.pdf]

nature portfolio

Corresponding author(s): Prof. Dr. Martin Beer
Prof. Dr. Anca Dorhoi

Last updated by author(s): Feb 3, 2023

Reporting Summary

Nature Portfolio wishes to improve the reproducibility of the work that we publish. This form provides structure for consistency and transparency in reporting. For further information on Nature Portfolio policies, see our Editorial Policies and the Editorial Policy Checklist.

Statistics

For all statistical analyses, confirm that the following items are present in the figure legend, table legend, main text, or Methods section.

n/a	Confirmed	
☐	☒	The exact sample size (n) for each experimental group/condition, given as a discrete number and unit of measurement
☐	☒	A statement on whether measurements were taken from distinct samples or whether the same sample was measured repeatedly
☐	☒	The statistical test(s) used AND whether they are one- or two-sided<br>*Only common tests should be described solely by name; describe more complex techniques in the Methods section.*
☒	☐	A description of all covariates tested
☒	☐	A description of any assumptions or corrections, such as tests of normality and adjustment for multiple comparisons
☒	☐	A full description of the statistical parameters including central tendency (e.g. means) or other basic estimates (e.g. regression coefficient) AND variation (e.g. standard deviation) or associated estimates of uncertainty (e.g. confidence intervals)
☒	☐	For null hypothesis testing, the test statistic (e.g. F, t, r) with confidence intervals, effect sizes, degrees of freedom and P value noted<br>*Give P values as exact values whenever suitable.*
☒	☐	For Bayesian analysis, information on the choice of priors and Markov chain Monte Carlo settings
☒	☐	For hierarchical and complex designs, identification of the appropriate level for tests and full reporting of outcomes
☒	☐	Estimates of effect sizes (e.g. Cohen's d, Pearson's r), indicating how they were calculated

Our web collection on statistics for biologists contains articles on many of the points above.

Software and code

Policy information about availability of computer code

Data collection	Flow cytometry data collection DIVA Version 9.0.1, BioRad CFX Maestro version 2, BioRender.com (2022)
Data analysis	Flow cytometry analysis FlowJo Version 10.5.3 and GraphPad Prism Version 8.4.2, Microsoft excel from Microsoft Office Professional Plus 2019 Version 1808

For manuscripts utilizing custom algorithms or software that are central to the research but not yet described in published literature, software must be made available to editors and reviewers. We strongly encourage code deposition in a community repository (e.g. GitHub). See the Nature Portfolio guidelines for submitting code & software for further information.

Data

Policy information about availability of data

All manuscripts must include a data availability statement. This statement should provide the following information, where applicable:
- Accession codes, unique identifiers, or web links for publicly available datasets
- A description of any restrictions on data availability
- For clinical datasets or third party data, please ensure that the statement adheres to our policy

The authors declare that the data supporting the findings of this study are available within the paper and its supplementary information files and are available from the corresponding authors upon reasonable request. All non-commercial materials generated during the current study are available from the corresponding authors under an MTA with Friedrich-Loeffler-Institut. Source data are provided with this paper. Accession codes: SARS-CoV-2 NCBI Reference Sequence NC_045512.2 with YP_009724390.1; SARS-CoV-2 B.1.617.2-lineage hCoV-19/Switzerland/BE-IFIK-918-4879/2021 (GISAID accession EPI_ISL_1760647) “Delta”; SARS-CoV-2 hCoV-19/

Germany/NW-RKI-I-0029/2020 B.1.351-lineage (GISAID accession EPI_ISL_803957) "Beta"; SARS-CoV-2 B.1.1.529 sublineage BA.1 "Omicron" FFM-ZAF0396/2021; GenBank accession: OM617939.1, GISAID accession EPI_ISL_6959868, sublineage BA.5 hCoV-19/South Africa/CERI-KRISP-K040013/2022, GISAID accession EPI_ISL_12268493 .

Human research participants

Policy information about studies involving human research participants and Sex and Gender in Research.

Reporting on sex and gender	Study did not involve human subjects
Population characteristics	Study did not involve human subjects
Recruitment	Study did not involve human subjects
Ethics oversight	Study did not involve human subjects

Note that full information on the approval of the study protocol must also be provided in the manuscript.

Field-specific reporting

Please select the one below that is the best fit for your research. If you are not sure, read the appropriate sections before making your selection.

[x] Life sciences [ ] Behavioural & social sciences [ ] Ecological, evolutionary & environmental sciences

For a reference copy of the document with all sections, see nature.com/documents/nr-reporting-summary-flat.pdf

Life sciences study design

All studies must disclose on these points even when the disclosure is negative.

Sample size	Assessment of animals in each group was based on a one-sided Fisher exact test with a significance level of 5% and a power of 80% (Power 1-ß = 80%) to allow detection of a significant reduction of the viral load by 50% between Sham and immunized groups.
Data exclusions	No data were excluded from the analyses.
Replication	Multiple animals were used to repeat each experiment. All data and the accompanying n-numbers are provided. Elisa-tests and RT-qPCR analyses were replicated once. VNTs were successfully assessed in triplicate.
Randomization	Animals were randomly allocated to treatment.
Blinding	No blinding to treatment allocation was used. This was a non-clinical study involving rats and mice, with the data collection and analyses relying on objective measures.

Reporting for specific materials, systems and methods

We require information from authors about some types of materials, experimental systems and methods used in many studies. Here, indicate whether each material, system or method listed is relevant to your study. If you are not sure if a list item applies to your research, read the appropriate section before selecting a response.

Materials & experimental systems

n/a	Involved in the study	
[ ]	[x]	Antibodies
[ ]	[x]	Eukaryotic cell lines
[x]	[ ]	Palaeontology and archaeology
[ ]	[x]	Animals and other organisms
[x]	[ ]	Clinical data
[x]	[ ]	Dual use research of concern

Methods

n/a	Involved in the study	
[x]	[ ]	ChIP-seq
[ ]	[x]	Flow cytometry
[x]	[ ]	MRI-based neuroimaging

Antibodies

Antibodies used	All details of antibodies used for this study are provided in the Supplementary Table S4.

Validation	All antibodies were validated using primary mouse splenocytes which were untreated or stimulated with PMA/Ionomycin. The dilution factor was determined by titration experiments with serial dilutions of the antibody (1:20 - 1:640). Dilution with the highest ratio of postive to negative population MFI was used for experiments and is provided in table S4. Cell populations were considered positive for a specific antibody staining by setting gates based on fluorescence minus one (FMO) controls. The following antibodies were validated by and purchased from Biolegend GmbH: anti mouse CD45 Alexa700, Isotype Rat IgG2b, k, Clone: 30-F11, Catalog: 103127 (https://www.biolegend.com/en-us/products/alexa-fluor-700-anti-mouse-cd45-antibody-3407?GroupID=BLG6833), Dilution factor: 3µg in 100µl PBS per mouse of CD45 Alexa 700 were administred intravenously (CD45i.v.). anti mouse CD3 APC/Cyanine7, Isotype Rat IgG2b, κ, Clone: 17A2, Catalog: 100221 (https://www.biolegend.com/en-us/products/apc-cyanine7-anti-mouse-cd3-antibody-6068), Dilution factor: 1:100. anti mouse CD4 Brilliant Violet 650™, Isotype Rat IgG2a, κ, Clone: RM4-5, Catalog: 100545 (https://www.biolegend.com/en-us/products/brilliant-violet-650-anti-mouse-cd4-antibody-7634), Dilution factor: 1:150. anti mouse CD8a Brilliant Violet 785™, Isotype Rat IgG2a, κ, Clone: 53-6.7, Catalog: 100749 (https://www.biolegend.com/en-us/products/brilliant-violet-785-anti-mouse-cd8a-antibody-7957), Dilution factor: 1:100. anti mouse TCR γ/δ Brilliant Violet 510™, Isotype Armenian Hamster IgG, Clone: MEL-14, Catalog: 118131 (https://www.biolegend.com/en-us/products/brilliant-violet-510-anti-mouse-tcr-gamma-delta-antibody-9740), Dilution factor: 1:50. anti mouse CD62L Brilliant Violet 605™, Isotype Rat IgG2a, κ, Clone: GL3, Catalog: 104437 (https://www.biolegend.com/en-us/products/brilliant-violet-605-anti-mouse-cd62l-antibody-7687), Dilution factor: 1:100. anti mouse CD103 Brilliant Violet 711™, Isotype Armenian Hamster IgG, Clone: 2E7, Catalog: 1121435 (https://www.biolegend.com/en-us/products/brilliant-violet-711-anti-mouse-cd103-antibody-14411), Dilution factor: 1:100. anti mouse CD95 APC, Isotype Rat IgG1, κ, Clone: SA367H8, Catalog: 152603 (https://www.biolegend.com/en-us/products/apc-anti-mouse-cd95-fas-antibody-13906), Dilution factor: 1:150. anti mouse CD44 PE, Isotype Rat IgG2b, κ, Clone: IM7, Catalog: 103023 (https://www.biolegend.com/en-us/products/pe-anti-mouse-human-cd44-antibody-2206), Dilution factor: 1:150. anti mouse KLRG1 Brilliant Violet 421™, Isotype Syrian Hamster IgG, Clone: 2F1/KLRG1, Catalog: 138413 (https://www.biolegend.com/en-us/products/brilliant-violet-421-anti-mouse-human-klrg1-mafa-antibody-7528), Dilution factor: 1:100. anti mouse CD183/ CXCR3 PE/ Cy7, Isotype Armenian Hamster IgG, Clone: CXCR3-173, Catalog: 126515 (https://www.biolegend.com/en-us/products/pe-cyanine7-anti-mouse-cd183-cxcr3-antibody-6169), Dilution factor: 1:100. anti mouse CD69 FITC, Isotype Armenian Hamster IgG, Clone: H1.2F3, Catalog: 104505 (https://www.biolegend.com/en-us/products/fitc-anti-mouse-cd69-antibody-264), Dilution factor: 1:100. anti mouse PD-1 PE-Dazzle 594, Isotype Rat IgG2b, κ, Clone: RMP1-30, Catalog: 109115 (https://www.biolegend.com/en-us/products/pe-dazzle-594-anti-mouse-cd279-pd-1-antibody-12479), Dilution factor: 1:100. anti mouse CD4 FITC, Isotype Rat IgG2a, κ, Clone: RM4-5, Catalog: 100509 (https://www.biolegend.com/en-us/products/fitc-anti-mouse-cd4-antibody-480), Dilution factor: 1:100. anti mouse T-bet Brilliant Violet 711™, Mouse IgG1, κ, Clone: 4B10 Catalog: 644819 (https://www.biolegend.com/en-us/products/brilliant-violet-711-anti-t-bet-antibody-7952), Dilution factor: 1:100. anti mouse IFN-γ Brilliant Violet 605™, Rat IgG1, κ, Clone: XMG1.2 Catalog: 505839 (https://www.biolegend.com/en-us/products/brilliant-violet-605-anti-mouse-ifn-gamma-antibody-8114), Dilution factor: 1:150. anti mouse IL-17A Brilliant Violet 421™, Rat IgG1, κ, Clone: TC11-18H10.1 Catalog: 506925 (https://www.biolegend.com/en-us/products/brilliant-violet-421-anti-mouse-il-17a-antibody-7223), Dilution factor: 1:100. anti mouse IL-10 PE-Cy7, Rat IgG2b, κ, Clone: JES516E3 Catalog: 505025 (https://www.biolegend.com/en-us/products/pe-cyanine7-anti-mouse-il-10-antibody-7773), Dilution factor: 1:50. anti mouse Granzyme B Alexa Fluor® 647, Mouse IgG1, κ, Clone: GB11 Catalog: 515405 (https://www.biolegend.com/en-us/products/alexa-fluor-647-anti-human-mouse-granzyme-b-antibody-6067), Dilution factor: 1:100. The following antibody was validated by and purchased from BD bioscience : anti mouse RORγT PE-CF594, Mouse IgG2a, Clone: Q31-378 Catalog: 515405 (https://www.bdbiosciences.com/en-us/products/reagents/flow-cytometry-reagents/research-reagents/single-color-antibodies-ruo/pe-cf594-mouse-anti-mouse-ror-t.562684), Dilution factor: 1:100. The following antibody was validated by and purchased from ThermoFisher: anti mouse FoxP3 PE-Cy5.5, Rat / IgG2a, κ, Clone: FJK-16s Catalog: 35-5773-80 (https://www.thermofisher.com/antibody/product/FOXP3-Antibody-clone-FJK-16s-Monoclonal/35-5773-82), Dilution factor: 1:50.

Eukaryotic cell lines

Policy information about cell lines and Sex and Gender in Research

Cell line source(s)	For generation of virus stocks: Vero E6 (Collection of Cell Lines in Veterinary Medicine 0929; CCLV FLI Insel Riems, Germany, originating from ATCC Vero C1008)
Authentication	The cell line was not in house authenticated.
Mycoplasma contamination	All cell lines were quality screened and were tested to ensure they were free of mycoplasma.
Commonly misidentified lines (See ICLAC register)	No misidentified cell lines were used.

Animals and other research organisms

Policy information about studies involving animals; ARRIVE guidelines recommended for reporting animal research, and Sex and Gender in Research

Laboratory animals	K18-hACE2 transgenic mice (8-10 weeks old at the time of vaccination/females) and Wistar rats (7-8 weeks old at the time of vaccination/females). Mice were maintained at 20–22°C and a relative humidity of 45±10% on a 12h light/dark cycle, fed with commercial rodent chow (Ssniff, Soest, Germany), and provided with tap water ad libitum. Rats were were kept in Macrolon Type IV cages on 12h light/dark cycle, at 20-22°C and a relative humidity of 45-65%, and had unlimited access to standard diet (Ssniff R/M, Soest, Germany) and water.
Wild animals	The study did not involve wild animals.
Reporting on sex	Only female mice/rats were used for this study. Male mice are more aggressive than female mice and increase the biosafety risk in the BSL3 during high titer intranasal challenge with virulent SARS-CoV-2. In previous rat vaccination studies no variability between sexes was observed.

Field-collected samples	The study did not involve samples collected from the field.
Ethics oversight	The animal experiments were evaluated and approved by the ethics committee of the State Office of Agriculture, Food safety, and Fishery in Mecklenburg – Western Pomerania (LALLF M-V: LVL MV/TSD/7221.3-1-055/20) and the State Office for Occupational Safety, Consumer Protection and Health in Brandenburg (LAVG: 2347-5-2021). All procedures using SARS-CoV-2 were carried out in approved biosafety level 3 (BSL3) facilities.

Note that full information on the approval of the study protocol must also be provided in the manuscript.

Flow Cytometry

Plots

Confirm that:

- [x] The axis labels state the marker and fluorochrome used (e.g. CD4-FITC).
- [x] The axis scales are clearly visible. Include numbers along axes only for bottom left plot of group (a 'group' is an analysis of identical markers).
- [x] All plots are contour plots with outliers or pseudocolor plots.
- [x] A numerical value for number of cells or percentage (with statistics) is provided.

Methodology

Sample preparation	Mouse spleen and lung tissue were harvested and kept at 4°C before either mechanical disruption (spleen) or mechanical disruption followed by enzymatic digestion (lung: 175 µg/ml LiberaseTM [Roche] and 0.1 mg/ml DNase I in serum-free medium) to generate single cell suspensions. Erythrocyte lysis in both suspensions was performed using 1 x red blood cell (RBC) lysis buffer (BioLegend) before determining cell counts. After an additional washing step, cell surface receptor staining began with Zombie UV™ Fixable dye (1:100; BioLegend) for 20 minutes in the dark at 4°C followed by two washing steps. Unspecific antibody binding was blocked with TruStain FcX (anti-mouse CD16/32) solution for 5 minutes at 4°C before adding freshly prepared antibody cocktails. Cells were incubated with surface antibodies for 20 minutes at 4°C in the dark, followed by two washing steps before fixation for 30 minutes at room temperature with 4% paraformaldehyde (PFA). SARS-CoV-2 S-peptide specific responses were investigated by culturing single cell suspensions in the presence of 0.5 µg/ ml PepMix™ SARS-CoV-2 (JPT) for 15 hours and brefeldin A (BioLegend) for an additional 4 hours. For intracellular antibody staining cells were washed twice before fixated with intracellular fixation buffer (eBioscience, Foxp3/Transcription Factor Staining Buffer Set) and washed with 1 x permeabilization buffer followed by incubation of antibody cocktails diluted in 1 x permeabilization buffer.
Instrument	BD FACS LSRFortessa Serial Nr: R649225B8012
Software	For collection of Data on the BD instrument DIVA 9.0.1 was used. For analysis fcs files were exported and compensated files were analyzed using FlowJo Version 10.5.3
Cell population abundance	For this project we analyzed T cells in response to vaccination and challenge with SARS-CoV-2. Lymphocytes (B and T cells) had the highest abundance of all leukocytes in the spleen. In the lung 100-10000 T cells of all events were acquired. 2x10^6 viable lung or spleen cells were stained and resuspended in 200µl. The entire volume was acquired during sample acquistion.
Gating strategy	Lymphocytes were identified by SSC-A vs FSC-A and lymphocyte doublets were excluded by FSC-H/FSC-A and SSC-W/SSC-H. Dead cells were eliminated using a fixable live/dead viability dye (UV zombie). To distinguish lung parenchymal (CD45iv-) from vascular (CD45iv+) CD3+ T cells, 3µg anti-mouse CD45 antibody was injected (i.v.) for 3 minutes during lethal anesthesia. CD3+ T cells were further analysed by exclusion of γδTCR+ cells before gating on CD8+ cytotoxic T cells and CD4+ helper T cells. CD4+ and CD8+ T cell subsets were further analysed for the frequency of CXCR3+ T cells or TRM cells defined as CD45iv-CD3+γδTCR-CD8+CD44highCD62L-CD103+CD69+. All gates were based on FMOs (fluorescent minus one). For SARS-CoV-2 specific T cells the unstimulated samples served as a control and to set the gate for SARS-CoV-2 specific responses after stimulation with peptide pools.

- [x] Tick this box to confirm that a figure exemplifying the gating strategy is provided in the Supplementary Information.
